# COVID-19 mortality among international migrants in Brazil: spatio-temporal analysis, 2020-2022

**DOI:** 10.1590/S2237-96222024V33E2024631.EN

**Published:** 2025-01-27

**Authors:** João Roberto Cavalcante, Anete Trajman, Eduardo Faerstein

**Affiliations:** 1 Universidade do Estado do Rio de Janeiro, Departamento de Epidemiologia, Rio de Janeiro, RJ, Brazil; 2 Universidade Federal do Rio de Janeiro, Departamento de Clínica Médica, Rio de Janeiro, RJ, Brazil; 3 McGill University, McGill International TB Centre, Montreal, Canadá; 4 Rede Brasileira de Pesquisa em Tuberculose, Brazil

**Keywords:** Migrantes, COVID-19, Mortalidad, Epidemiología, Salud de Los Migrantes, Brasil, Transients and migrants, COVID-19, Mortality, Epidemiology, Global Health, Brazil

## Abstract

**Objective:**

To describe the mortality profile and analyze the spatiotemporal distribution of COVID-19 mortality among international migrants residing in Brazil from 2020 to 2022.

**Methods:**

This is a descriptive and ecological cross-sectional study using secondary data. Absolute and relative frequencies of the sociodemographic profile and mortality coefficients (MCs) were analyzed. Excess risk and global and local spatial autocorrelation were calculated.

**Results:**

A total of 7,737 deaths were recorded during the period, with the highest frequency in 2021 (3,952). Brazil’s overall MC was 515/100,000, with higher MCs in the Southeast (751/100,000) and Midwest (525/100,000) macroregions. The predominant death profile was for males (5,041); those aged ≥ 81 years (3,612); those of White race/skin color (5,685); married (3,406); born in Portugal (2,437). Global spatial autocorrelation was identified in 2022, and local spatial autocorrelation throughout the period.

**Conclusion:**

The high MC indicates a need for health policy interventions in regions with high migrant population concentrations.

## Study contributions

**Main results:**

The COVID-19 mortality coefficient among migrants in Brazil was 515/100,000, and higher in the Southeast and Midwest regions of the country. The highest number of deaths occurred in 2021, among White, married men, over 81 years old, mainly from Portugal.

**Implications for services:**

This study recommends the creation of policies for international migrants in Brazil, such as prioritizing vaccination campaigns, translation services and integration strategies to improve access to the health system, in order to reduce deaths.

**Perspectives:**

The Ministry of Health needs to create the National Health Policy for Migrants, Refugees and Stateless Persons, and the country of birth/nationality/place of birth variable needs to become a required field for filling out on health information systems.

## RESUMEN

**Objetivo:**

Describir el perfil de muertes, analizar la distribución espacio-temporal de la mortalidad por Covid-19 en migrantes internacionales residentes en Brasil durante 2020-2022.

**Métodos:**

Estudio descriptivo, ecológico y transversal, con datos secundarios. Se analizaron las frecuencias absolutas y relativas del perfil sociodemográfico y los coeficientes de mortalidad (CM). Se calcularon el exceso de riesgo y la autocorrelación espacial global y local.

**Resultados:**

Se registraron 7.737 muertes en el período, con mayor frecuencia en 2021 (3.952). El CM de Brasil fue de 515/100.000, con CM más altos en el Sudeste (751/100.000) y Centro-Oeste (525/100.000). El perfil predominante de muertes fueron hombres (5.041); edad ≥ 81 años (3.612); raza/color de piel blanca (5.685); casados (3.406); nacidos en Portugal (2.437). La autocorrelación espacial global se identificó en 2022 y la local en el período.

**Conclusión:**

El alto CM indica la necesidad de intervenciones en políticas de salud en regiones con alta concentración de población migrante.

## INTRODUCTION

Contemporary migratory processes are a global phenomenon, and have been taking on specific characteristics on the various continents.^1^ Spurred on by the quest for better living conditions, health services, job opportunities or refuge, they are a complex dimension of current population dynamics.^1^ At the global level, there are estimated to be 281 million international migrants, counted as at the end of 2020 – the highest numbers ever recorded.^2^ In Brazil, a country that has historically received and still receives substantial migratory influxes, the presence of the current 1.6 million migrants resident in the country is an important component of our demographic dynamics.^3^

Historically, having recorded large influxes of Portuguese, Japanese, Italian, German migrants, as well as enslaved people from Nigeria, Côte d’Ivoire, Congo, Angola and Mozambique, Brazil currently records massive influxes of migrants coming mainly from Latin American and Central American countries, such as Venezuelans and Haitians.^4,5^

International migrants in general are subject to socioeconomic, environmental, cultural and psychosocial challenges that impact their health/illness process before, during and after migration.^6,7^ The most vulnerable are those who migrate involuntarily, such as refugees and stateless people.^6^ In Brazil, the main health problems of international migrants are chronic diseases, mental disorders and violence, unlike other host countries, such as those in Europe, where, for the most part, the main health problems recorded relate to communicable diseases.^7^

There is, however, scarcity of data on the health profile of this population.^8^ Although some countries have robust health information systems, which have recorded health and disease data on the resident population for decades, these systems are insufficient in relation to migrants, making make them invisible, which makes it difficult to implement specific health policies that protect them.^8^

In Brazil, only the Mortality Information System (*Sistema de Informação sobre Mortalidade* - SIM) has a field for recording country of birth of people who die here.^9^ Although SIM records have been available since 1979, as far as we know, data on migrants have never been analyzed and released nationally.^8^ A significant part of this difficulty arises from the need to calculate the total number of people at risk (denominators) in order to calculate mortality coefficients (MCs), thus allowing comparison between different places of residence or countries of birth.^8^ This was no different during the COVID-19 pandemic. Therefore, the number of COVID-19 cases and deaths among international migrants living in Brazil is still unknown. Perhaps for this reason, migrants were not considered a priority group by the country’s national COVID-19 immunization plan.^10^

The objective of this study was to describe the mortality profile and analyze the spatiotemporal distribution of COVID-19 mortality among international migrants residing in Brazil from 2020 to 2022.

## METHODS

A descriptive and ecological cross-sectional study was carried out, using secondary data, to assess the mortality profile and the spatio-temporal distribution of COVID-19 MCs among international migrants residing in Brazil. The units of analysis of the ecological study were countries of birth, Federative Units and macroregions of residence in Brazil.

Brazil has 5,570 municipalities, distributed across 26 states and the Federal District, which make up the country’s five macroregions. It is the fifth largest country in the world in terms of territorial area (8,547,403 km²).^11,12^ Brazil had an estimated total population of 203,080,756 inhabitants in 2022 – the world’s seventh largest population –, with population density of 23.8 inhabitants per km².^11,12^

The study included COVID-19 deaths recorded between 2020 and 2022.^13^ The year 2023 was not included, as the available data was incomplete.^9^ We used the “PLACE OF BIRTH” variable in order to separate international migrants from Brazilian nationals in the SIM databases.^9^ COVID-19 deaths were considered to be records held on the SIM database, which can be accessed via the Brazilian Ministry of Health *Opendatasus* website, for which underlying cause of death was ICD-10 code B34.2 (coronavirus infection, unspecified site) and code J98.8 (other specified respiratory disorders).^9^ Code J98.8 was used by health surveillance teams in Brazil when it was not possible to close cases of deaths as being due to “severe acute respiratory syndrome (SARS), unspecified”, such as COVID-19, due-the large volume of work during the pandemic.^9,14^

 Taking these data, absolute and relative frequencies were calculated in relation to year of death (2020, 2021 and 2022), sex (male, female and unknown), age group (< 1, 1-10, 11-20, 21-30, 31-40, 41-50, 51-60, 61-70, 71-80, ≥ 81, unknown), race/skin color (White, Black, mixed race, Asian, Indigenous and unknown) and marital status (single, married, widowed, separated/divorced, civil partnership and unknown). In order to calculate MCs per 100,000 inhabitants, it was necessary to prepare denominators for the population of migrants residing in Brazil, by country of birth, state and macroregion of residence in Brazil during the study period (2020 to 2022). This meant calculating this population from 2011 to 2022, even if only using the population between 2020 and 2022 as denominators, as the last calculated data on the population of international migrants in Brazil was for the year 2010. Thus, the open data used to calculate following years were those of the 2010 Demographic Census, the Brazilian Institute of Geography and Statistics (*Instituto Brasileiro de Geografia e Estatística* - IBGE), the National Migration Registration System (*Sistema de Registro Nacional Migratório* - SISMIGRA), the Ministry of Justice (2011 to 2022), in addition to SIM death data (2011 to 2022).^9,15,16^ Based on these data, it was possible to calculate, for the first time, the populations of international migrants residing in Brazil from 2020 to 2022. The formula for calculating these populations for the year 2011 was as follows:


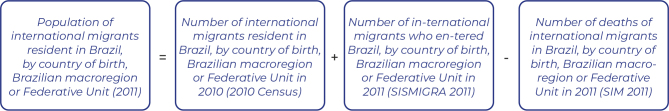


For the populations from 2012 to 2022, it was no longer necessary to use the 2010 Census separately, as it was already incorporated into the population calculated for the year 2011. The formula used to calculate the populations of the following years was as follows:


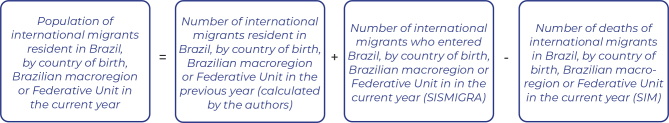


The mid-period population (MPP) was used in the MC denominator, whereby July 2 was taken to be the mid-period of each year, as shown in the formula described below:^17^


$$\text{MC}=\frac{\text{Number of deaths}}{\text{Mid-period population}}\times 100,000$$

The tables containing the MCs were presented in heatmap format, in which the lower values in each column have a lighter color and the higher values appear in a darker color, in order to facilitate visualization. The COVID-19 MCs per Federative Unit were also used to calculate excess risk, the Global Moran index (Moran’s I), and the local indicators of spatial association (LISA).^18^

Excess risk was presented in thematic maps and calculated by comparing the MCs for COVID-19 in different Federative Units in Brazil with the national MC, observing whether the MCs in each Federative Unit were higher or lower than the national MC.^18^ Moran’s I and LISA, in turn, analyze global and local spatial autocorrelation, with the aim of identifying patterns in the spatial distribution of the indicators presented.^18^ In this study, they were used to identify clusters of deaths in the Brazilian Federative Units and their statistical significance during the study period.^18^ To calculate Moran’s I, we used a matrix of spatial weights that defines the neighborhood relationships between the Federative Units and applied tests of statistical significance to evaluate the null hypothesis of absence of spatial autocorrelation.^18^ For LISA, in turn, we disaggregated the global analysis, identifying areas with high and low MCs. This procedure includes the calculation of LISA values for each Federative Unit and the evaluation of their significance through permutation tests, thus enabling detection of local spatial patterns during the study period.^18^

The high-high, low-low, high-low, and low-high quadrants were used to classify local spatial autocorrelation.^18^ The high-high quadrant indicates areas with high values neighboring other areas with high values; low-low indicates areas with low values neighboring other areas with low values; high-low indicates areas with high values neighboring areas with low values; and low-high indicates areas with low values neighboring areas with high values.^18^ These quadrants were presented using scatterplots and cluster maps.^18^ We used R Studio 3.3.0+ software for data manipulation and analysis, and we used the Quantum Geographic Information System (QGIS) version 3.24 and GeoDa version 1.22 for spatial analysis.

As this is a study using secondary, aggregated and freely accessible data, there was no need for approval by a Research Ethics Committee, in accordance with National Research Ethics Council Resolution No. 510/2016. The databases we analyzed were public and anonymized, as provided for by the General Data Protection Law (Law No. 13709/2018).^19,20^

## RESULTS

Between 2020 and 2022, 7,737 COVID-19 deaths were recorded for international migrants residing in Brazil, the majority of which occurred in 2021 (Table 1). The majority of deaths were in males and elderly people, with the average age at death being 76.3 years. The predominant race/skin color was White, followed by Asian. Most individuals were married or widowed (Table 1).

**Table 1:** Sociodemographic profile of COVID-19 deaths of international migrants resident in Brazil, between 2020 and 2022

**Sociodemographic characteristics**	**2020**		**2021**		**2022**		**2020-2022 (Full period)**	
	**N**	**%**	**N**	**%**	**N**	**%**	**N**	**%**
Year of death	2,865	37.0	3,952	51.1	920	11.9	7,737	100
**Sex**								
Male	1,871	65.3	2,617	66.2	553	60.1	5,041	71.5
Female	993	34.7	1,333	33.7	367	39.9	2,693	38.2
Unknown	1	0.03	2	0.1	0	0.0	3	0.0
**Age Group (years)**								
< 1	0	0.0	2	0.1	0	0.0	2	0.0
1 a 10	1	0.0	1	0.0	2	0.2	4	0.1
11 a 20	3	0.1	4	0.1	1	0.1	8	0.1
21 a 30	15	0.5	39	1.0	2	0.2	56	0.7
31 a 40	54	1.9	131	3.3	11	1.2	196	2.5
41 a 50	73	2.5	244	6.2	11	1.2	328	4.2
51 a 60	160	5.6	357	9.0	15	1.6	532	6.9
61 a 70	376	13.1	655	16.6	69	7.5	1,100	14.2
71 a 80	695	24.3	1,025	25.9	179	19.5	1,899	24.5
≥ 81	1,488	51.9	1,494	37.8	630	68.5	3,612	46.7
Unknown	0	0.0	0	0.0	0	0.0	0	0.0
**Race/skin color**								
White	2,127	74.2	2,845	72.0	713	77.5	5,685	73.5
Asian	329	11.5	630	15.9	70	7.6	1,029	13.3
Mixed race	279	9.7	269	6.8	105	11.4	653	8.4
Black	68	2.4	101	2.6	14	1.5	183	2.4
Indigenous	15	0.5	19	0.5	4	0.4	38	0.5
Unknown	47	1.6	88	2.2	14	1.5	149	1.9
**Marital status**								
Single	301	10.5	571	14.4	100	10.9	972	12.6
Married	1,229	42.9	1,818	46.0	359	39.0	3,406	44.0
Widowed	942	32.9	1,014	25.7	359	39.0	2,315	29.9
Separated/divorced	174	6.1	261	6.6	53	5.8	488	6.3
Civil partnership	86	3.0	117	3.0	17	1.8	220	2.8
Unknown	133	4.6	171	4.3	32	3.5	336	4.3

Regarding country of birth, the majority were from Portugal, followed by Italy, Japan, Bolivia, Spain, Venezuela, Germany, Paraguay, Peru, Lebanon, among others (Table 2). The highest MCs due to COVID-19 related to international migrants born in Lithuania, followed by Jordan, Portugal, Hungary, Lebanon, Egypt, Poland, Japan, Greece, Italy, among others (Table 2).

**Table 2:** Distribution of COVID-19 deaths of international migrants resident in Brazil, by country of birth, between 2020 and 2022

Country of birth	2020				2021				2022				2020-2022 (Full period)			
	N	%	MPP	MC	N	%	MPP	MC	N	%	MPP	MC	N	%	MPP	MC
Angola	14	0.5	15,961.5	87.7	20	0.5	16,834.5	118.8	1	0.1	18,597.5	5.4	35	0.5	16,834.5	207.9
Argentina	62	2.2	64,561.0	96.0	109	2.8	67,330.5	161.9	17	1.8	71,364.5	23.8	188	2.4	67,330.5	279.2
Austria	7	0.2	2,690.5	260.2	15	0.4	2,696.5	556.3	5	0.5	2,746.0	182.1	27	0.3	2,696.5	1,001.3
Belgium	4	0.1	4,143.5	96.5	8	0.2	4,202.0	190.4	0	0.0	4,287.5	0.0	12	0.2	4,202.0	285.6
Bolivia	177	6.2	105,624.0	167.6	268	6.8	109,777.0	244.1	38	4.1	115,422.5	32.9	483	6.2	109,777.0	440.0
Chile	63	2.2	22,885.5	275.3	104	2.6	23,307.0	446.2	17	1.8	23,846.5	71.3	184	2.4	23,307.0	789.5
China	54	1.9	50,808.5	106.3	105	2.7	51,552.0	203.7	16	1.7	52,524.0	30.5	175	2.3	51,552.0	339.5
Colombia	24	0.8	69,417.0	34.6	53	1.3	73,210.5	72.4	6	0.7	77,639.0	7.7	83	1.1	73,210.5	113.4
Cuba	10	0.3	31,040.0	32.2	29	0.7	31,729.5	91.4	1	0.1	33,718.0	3.0	40	0.5	31,729.5	126.1
Ecuador	6	0.2	8,602.5	69.7	7	0.2	9,094.5	77.0	1	0.1	9,976.5	10.0	14	0.2	9,094.5	153.9
Egypt	19	0.7	1,997.0	951.4	26	0.7	2,008.0	1,294.8	6	0.7	2,028.5	29.8	51	0.7	2,008.0	2,539.8
France	16	0.6	32,583.0	49.1	27	0.7	33,397.0	80.8	7	0.8	34,673.5	20.2	50	0.6	33,397.0	149.7
Germany	37	1.3	27,505.0	134.5	175	4.4	27,505.5	636.2	30	3.3	27,682.5	108.4	242	3.1	27,505.5	879.8
Grece	13	0.5	2,304.0	564.2	29	0.7	2,256.0	1,285.5	3	0.3	2,214.5	135.5	45	0.6	2,256.0	1,994.7
Guiana	4	0.1	1,045.5	382.6	12	0.3	1,065.0	1,126.8	1	0.1	1,120.0	89.3	17	0.2	1,065.0	1,596.2
Haiti	32	1.1	154,658.0	20.7	38	1.0	165,342.0	23.0	7	0.8	170,067.0	4.1	77	1.0	165,342.0	46.6
Hungary	7	0.2	679.5	1,030.2	11	0.3	668.0	1,646.7	2	0.2	657.0	304.4	20	0.3	668.0	2,994.0
Israel	6	0.2	1,515.0	396.0	9	0.2	1,532.0	587.5	3	0.3	1,538.5	195.0	18	0.2	1,532.0	1,174.9
Italy	274	9.6	39,473.0	694.1	338	8.6	38,617.0	875.3	81	8.8	37,981.5	213.3	693	9.0	38,617.0	1,794.5
Japan	274	9.6	30,555.5	896.7	252	6.4	29,160.0	864.2	120	13.0	28,046.0	427.9	646	8.3	29,160.0	2,215.4
Jordan	6	0.2	538.0	1,115.2	17	0.4	523.5	3,247.4	1	0.1	513.0	194.9	24	0.3	523.5	4,584.5
Lebanon	67	2.3	7,092.0	944.7	115	2.9	7,010.0	1,640.5	19	2.1	7,015.0	270.8	201	2.6	7,010.0	2,867.3
Lithuania	5	0.2	238.5	2,096.4	3	0.1	198.0	1,515.2	3	0.3	168.5	1,780.4	11	0.1	198.0	5,555.6
Mozambique	7	0.2	4,028.0	173.8	7	0.2	4,171.5	167.8	2	0.2	4,405.0	45.4	16	0.2	4,171.5	383.6
Netherlands	6	0.2	8,034.5	74.7	16	0.4	8,139.0	196.6	4	0.4	8,308.0	48.1	26	0.3	8,139.0	319.4
Nigeria	5	0.2	4,833.0	103.5	6	0.2	5,010.0	119.8	1	0.1	5,276.5	19.0	12	0.2	5,010.0	239.5
Paraguay	50	1.7	47,852.0	104.5	142	3.6	49,596.0	286.3	29	3.2	52,562.0	55.2	221	2.9	49,596.0	445.6
Peru	85	3.0	40,400.5	210.4	123	3.1	41,570.5	295.9	9	1.0	43,183.0	20.8	217	2.8	41,570.5	522.0
Poland	41	1.4	3,982.5	1,029.5	34	0.9	3,923.5	866.6	18	2.0	3,938.0	457.1	93	1.2	3,923.5	2,370.3
Portugal	1,005	35.1	86,271.0	1,164.9	1,132	28.6	81,119.5	1,395.5	300	32.6	76,359.5	392.9	2,437	31.5	81,119.5	3,004.2
Romania	17	0.6	2,502.0	679.5	12	0.3	2,486.5	482.6	7	0.8	2,499.5	280.1	36	0.5	2,486.5	1,447.8
Russia	7	0.2	4,773.5	146.6	5	0.1	5,090.5	98.2	3	0.3	5,624.5	53.3	15	0.2	5,090.5	294.7
South Korea	22	0.8	19,586.0	112.3	32	0.8	19,985.5	160.1	3	0.3	20,388.0	14.7	57	0.7	19,985.5	285.2
Spain	188	6.6	35,427.0	530.7	207	5.2	34,755.0	595.6	68	7.4	34,253.5	198.5	463	6.0	34,755.0	1,332.2
Switzerland	7	0.2	6,150.0	113.8	7	0.2	6,252.0	112.0	7	0.8	6,441.5	108.7	21	0.3	6,252.0	335.9
Syria	19	0.7	4,115.5	461.7	20	0.5	4,027.0	496.6	5	0.5	3,950.5	126.6	44	0.6	4,027.0	1,092.6
Turkey	2	0.1	1,452.0	137.7	7	0.2	1,537.0	455.4	1	0.1	1,678.5	59.6	10	0.1	1,537.0	650.6
United Kingdom	3	0.1	14,538.0	20.6	14	0.4	14,862.5	94.2	3	0.3	15,314.5	19.6	20	0.3	14,862.5	134.6
Uruguay	41	1.4	48,108.5	85.2	88	2.2	49,169.5	179.0	22	2.4	50,682.5	43.4	151	2.0	49,169.5	307.1
USA	8	0.3	51,346.5	15.6	25	0.6	52,823.0	47.3	10	1.1	54,898.5	18.2	43	0.6	52,823.0	81.4
Venezuela	95	3.3	221,963.5	42.8	205	5.2	272,560.0	75.2	23	2.5	349,267.5	6.6	323	4.2	272,560.0	118.5
Other	76	2.7	137,441.5	55.3	100	2.5	140,832.5	71.0	20	2.2	146,056.5	13.7	196	2.5	140,832.5	139.2
Total	2,865	100	1,418,723.5	199.6	3,952	100	1,496,927	260.9	920	100	1,608,915	56.5	7,737	100	1,496,927	516.9

Notes: N = absolute frequency of deaths % = relative frequency of deaths MPP = mid-period population MC = mortality coefficient USA = United States of America Color scale (from lowest values to highest values):
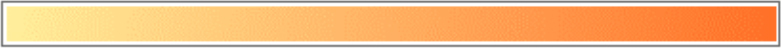

The overall MC was 515.6 deaths per 100,000 inhabitants. The Southeast macroregion had the highest number of deaths, followed by the Southern macroregion. Regarding MCs, the Southeast macroregion continued to stand out, followed by the Midwest macroregion. Although the highest number of deaths occurred in São Paulo and Rio de Janeiro, the highest MCs were found in Acre, followed by Goiás, São Paulo, Tocantins, among others (Table 3).

**Table 3:** Distribution of COVID-19 deaths of international migrants resident in Brazil, by Federative Unit and macroregion, between 2020 and 2022

Federative Units	2020				2021				2022				2020-2022 (Full period)			
	N	%	MPP	MC	N	%	MPP	MC	N	%	MPP	MC	N	%	MPP	MC
North	177	6.2	210,849.5	83.9	291	7.4	241,737.0	120.4	22	2.4	288,302.0	7.6	490	6.3	241,737.0	202.7
Acre	6	0.2	3,200.5	187.5	26	0.7	3,320.0	783.1	2	0.2	3,566.0	56.1	34	0.4	3,320.0	1,024.1
Amapá	2	0.1	2,053.0	97.4	2	0.1	2,139.0	93.5	0	0.0	2,243.5	0	4	0.1	2,139.0	187.0
Amazonas	68	2.4	61,797.0	110.0	104	2.6	68,717.0	151.3	4	0.4	77,110.0	5.2	176	2.3	68,717.0	256.1
Pará	22	0.8	10,801.0	203.7	28	0.7	11,274.0	248.4	4	0.4	11,927.5	33.5	54	0.7	11,274.0	479.0
Rondônia	24	0.8	9,730.0	246.7	29	0.7	10,521.5	275.6	5	0.5	11,659.5	42.9	58	0.7	10,521.5	551.3
Roraima	50	1.7	121,722.5	41.1	97	2.5	144,160.0	67.3	6	0.7	180,108.5	3.3	153	2.0	144,160.0	106.1
Tocantins	5	0.2	1,545.5	323.5	5	0.1	1,605.5	311.4	1	0.1	1,687.0	59.3	11	0.1	1,605.5	685.1
Northeast	94	3.3	111,290.0	84.5	186	4.7	115,096.0	161.6	41	4.5	119,999.5	34.2	321	4.1	115,096.0	278.9
Alagoas	3	0.1	3,214.5	93.3	4	0.1	3,370.5	118.7	2	0.2	3,578.0	55.9	9	0.1	3,370.5	267.0
Bahia	35	1.2	33,011.5	106.0	70	1.8	34,111.0	205.2	16	1.7	35,489.5	45.1	121	1.6	34,111.0	354.7
Ceará	17	0.6	25,333.0	67.1	33	0.8	26,061.0	126.6	4	0.4	27,177.5	14.7	54	0.7	26,061.0	207.2
Maranhão	4	0.1	7,239.0	55.3	14	0.4	7,497.0	186.7	1	0.1	7,808.5	12.8	19	0.2	7,497.0	253.4
Paraíba	3	0.1	6,490.5	46.2	9	0.2	6,705.5	134.2	1	0.1	6,990.5	14.3	13	0.2	6,705.5	193.9
Pernambuco	23	0.8	19,773.5	116.3	35	0.9	20,400.5	171.6	13	1.4	21,205.0	61.3	71	0.9	20,400.5	348.0
Piauí	1	0.0	2,676.0	37.4	3	0.1	2,809.0	106.8	0	0.0	2,941.5	0	4	0.1	2,809.0	142.4
Rio Grande do Norte	6	0.2	10,602.0	56.6	9	0.2	11,081.5	81.2	3	0.3	11,613.5	25.8	18	0.2	11,081.5	162.4
Sergipe	2	0.1	2,950.0	67.8	9	0.2	3,060.0	294.1	1	0,1	3,195.5	31.3	12	0.2	3,060.0	392.2
Midwest	97	3.4	87,989.5	110.2	346	8.8	93,679.5	369.3	49	5.3	100,918.0	48.6	492	6.4	93,679.5	525.2
Distrito Federal	31	1.1	30,672.5	101.1	56	1.4	31,815.5	176.0	12	1.3	33,289.5	36.0	99	1.3	31,815.5	311.2
Goiás	19	0.7	18,291.5	103.9	154	3.9	19,238.0	800.5	8	0.9	20,251.0	39.5	181	2.3	19,238.0	940.8
Mato Grosso	17	0.6	17,221.0	98.7	37	0.9	18,673.5	198.1	5	0.5	20,522.5	24.4	59	0.8	18,673.5	316.0
Mato Grosso do Sul	30	1.0	21,804.5	137.6	99	2.5	23,952.5	413.3	24	2.6	26,855.0	89.4	153	2.0	23,952.5	638.8
Southeast	2,275	79.4	731,416.5	311.0	2,651	67.1	746,130.5	355.3	682	74.1	769,095.5	88.7	5,608	72.5	746,130.5	751.6
Espírito Santo	14	0.5	13,405.5	104.4	17	0.4	13,779.5	123.4	6	0.7	14,233.5	42.2	37	0.5	13,779.5	268.5
Minas Gerais	32	1.1	56,649.5	56.5	109	2.8	59,054.0	184.6	30	3.3	62,209.0	48.2	171	2.2	59,054.0	289.6
Rio de Janeiro	646	22.5	167,520.0	385.6	722	18.3	168,631.5	428.2	144	15.7	171,568.5	83.9	1512	19.5	168,631.5	896.6
São Paulo	1,583	55.3	493,841.5	320.5	1,803	45.6	504,665.5	357.3	502	54.6	521,084.5	96.3	3,888	50.3	504,665.5	770.4
South	222	7.7	280.526,5	79.1	478	12.1	303,910.0	157.3	126	13.7	335,853.0	37.5	826	10.7	303,910.0	271.8
Paraná	107	3.7	99,427.0	107.6	273	6.9	108,051.5	252.7	75	8.2	119,965.5	62.5	455	5.9	108,051.5	421.1
Rio Grande do Sul	59	2.1	95,603.0	61.7	112	2.8	101,554.0	110.3	26	2.8	109,236.5	23.8	197	2.5	101,554.0	194.0
Santa Catarina	56	2.0	85,496.5	65.5	93	2.4	94,304.5	98.6	25	2.7	106,651.0	23.4	174	2.2	94,304.5	184.5
Brazil	2,865	100	1,422,072	201.5	3,952	100	1,500,553	263.4	920	100	1,614,168	57.0	7,737	100	1,500,553	515.6

Notes: N = absolute frequency of deaths % = relative frequency of deaths MPP = mid-period population MC = mortality coefficient Color scale (from lowest values to highest values):
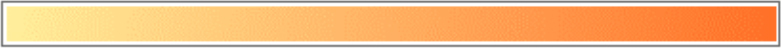

**Figure 1:**
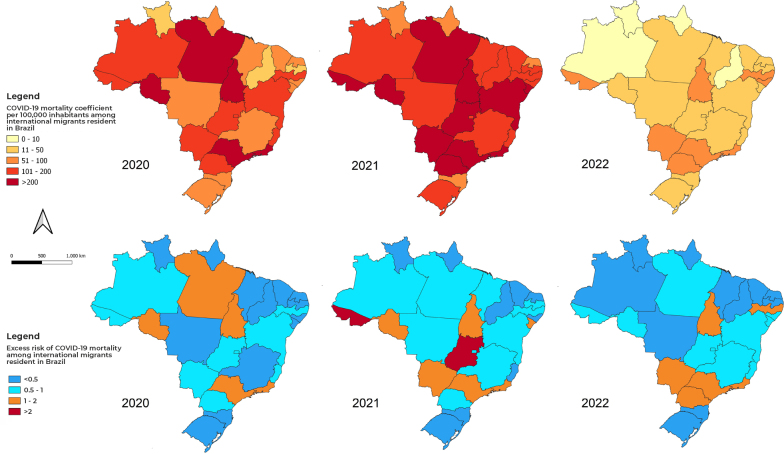
Coefficients and excess risk of COVID-19 mortality among international migrants resident in Brazil, by Federative Unit, between 2020 and 2022 (N = 7,737 deaths)

Excess risk of mortality in 2020 was higher in Pará, Rio de Janeiro, Rondônia, São Paulo and Tocantins, which had MCs up to twice as high as Brazil’s overall MC (Figure 1). In 2021, Acre and Goiás stand out, and in 2022, Mato Grosso do Sul, Paraná, Pernambuco, Rio de Janeiro, São Paulo and Tocantins stand out, with the MCs up to two times greater than the overall MC for Brazil. In 2022, no state had a MC more than double the overall MC for Brazil (Figure 1).

**Figure 2:**
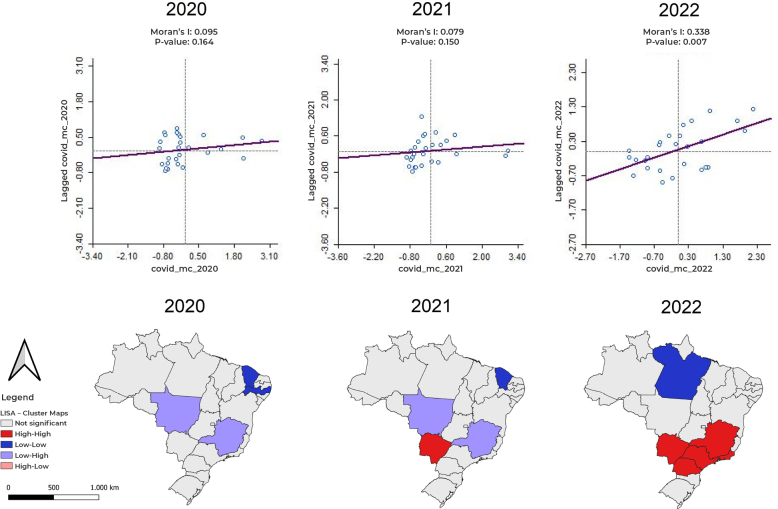
Global (Moran’s I) and local (LISA) spatial autocorrelation of COVID-19 mortality coefficients among international migrants resident in Brazil, by Federative Unit, between 2020 and 2022 (N = 7,737 deaths)

Moran’s I indicated global spatial autocorrelation in 2022, suggesting that COVID-19 deaths were related to each other and grouped in different states of the country, forming clusters (Moran’s I 0.338; p-value=0.007). LISA, in turn, indicated local spatial autocorrelation. Between 2020 and 2021, in Mato Grosso and Minas Gerais the MC changed from low to high values (low-high), which suggests a significant increase in COVID-19 mortality rates in these areas. Between 2021 and 2022, Mato Grosso do Sul and, specifically in 2022, the states of Paraná, Minas Gerais, Rio de Janeiro and São Paulo had high MCs that rose even further (high-high) (Figure 2).

## DISCUSSION

As far as it was possible to identify, this was the first national-level study to calculate the MCs of international migrants residing in Brazil. We found COVID-19 MCs almost twice as high among migrants than in the general population, suggesting a high risk of fatality in this population. The profile of international migrants residing in Brazil who died from COVID-19, between 2020 and 2022, was similar to that of the general population,^21^ with a higher number of deaths in 2021, among elderly men, coming mainly from European countries and concentrated in the Southeast and Southern macroregions.

Brazil had high COVID-19 mortality in absolute numbers – the second highest in the world, with a peak in deaths during transmission of the Gamma variant in the first half of 2021.^22^ The higher number of deaths among the elderly is not surprising; advanced age was soon recognized as a risk factor for high COVID-19 mortality in the general population.^23^ There were mass international migrations from Portugal, Italy and Japan to Brazil during the first half of the 20th century,^1,8^ and these migrants are in the oldest age group, which may explain the origin of migrants who died most due to COVID-19. In studies carried out in France and Spain, COVID-19 mortality was higher among elderly non-European migrants.^24,25^

In another unique study found on mortality of international migrants, conducted in Italy and published in 2020, the majority of migrants were born mainly in Albania, France, India, Libya and Romania. These migrants had a sociodemographic profile similar to that of the national population, the only difference being that the migrants who died the most, even if elderly, were younger than the population born in the country.^26^

The Southeast and Southern macroregions concentrated 83.2% of the total deaths, 75.7% of them in the states of São Paulo, Rio de Janeiro and Paraná. These locations also concentrate the majority of the international migrant population residing in Brazil.^16^ However, the highest MCs were found in the Southeast and Midwest macroregions. The states of Acre and Goiás stand out, especially in 2021. Acre has received a variety of international migrants in recent years, mainly Haitians.^5^ The high MC in Goiás can be explained by the high number of elderly international migrants who arrived there from Germany during the 20th century, to work on plantations.^27^

Currently, even with the provisions of the 1988 Federal Constitution, the 1990 Organic Health Law and the 2017 Migration Law, which guarantees access to health and protection for international migrants in Brazil, this population still faces difficulty in receiving care and treatment.^7^ Some positive movements have been made regarding the health of international migrants in recent years in Brazil, with the country’s return to the Global Compact for Safe, Orderly and Regular Migration, and the publication, by the Ministry of Health, of the ordinance intended to set up a working group to contribute to the creation of the future National Health Policy for Migrants, Refugees and Stateless Persons.^28,29^

Our study has some limitations. As there are no official records of the profile of the migrant population residing in Brazil, it was not possible to compare COVID-19 MCs with the general profile of migrants in the country, to estimate whether the greater number of deaths was due to the larger population of these migrants or greater risk in these subpopulations. In the context of COVID-19, this population was made invisible in the E-SUS *Notifica* System and in the Influenza Epidemiological Surveillance Information System (*Sistema de Informações sobre Vigilância Epidemiológica da Gripe* - SIVEP-Gripe), because, in both systems, filling out the “country of birth, “place of birth” or “nationality” variables is not mandatory. In the case of the E-SUS *Notifica* System, another factor causes this data to be lost, as the variable “country of birth” can only be completed in cases in which where the person does not have a Personal Taxpayer Registration Number (*Cadastro de Pessoa Física* - CPF). However, documented international migrants can obtain their CPF in Brazil, which makes Mortality Information System (SIM) the only health information system with data on these individuals.^8^

The IBGE Census due to take place in 2020 was not carried out because of the pandemic, and the results of the 2022 Census on the migrant population had not been released when we concluded this research, which made more precise estimates of the denominators for calculating MCs unfeasible. The lack of data, for example, on undocumented international migrants in Brazil, and incomplete SISMIGRA data (2011-2022) made it impossible to create more precise denominators that considered the sex and age range of these populations, which are important for better evaluation and comparison of MCs. It was also not possible to perform linear interpolation for migrant populations by municipality, due to their constantly changing their place of residence between neighboring cities. We tried to minimize this limitation, by carrying out analyses by macroregion and Federative Units, as they are larger analysis units, which reduce the possibility of bias.

We conclude that the invisibility of international migrants in national health statistics during the COVID-19 pandemic reflects a relevant gap in the inclusion of this group in public health policies. Brazil urgently needs to create health policies for international migrants, including them as a vulnerable and priority population in emergency plans, which favor these people’s access to health services, medicines and supplies such as vaccines, thus guaranteeing the improvement of their long-term health conditions.
