# Supplementary material for: COVID-19 mortality among international migrants in Brazil: spatio-temporal analysis, 2020-2022
Source: Epidemiol Serv Saude. 2025 Jan 27;33:e2024631. doi: 10.1590/S2237-96222024V33E2024631.EN (PMC11776071; doi:10.1590/S2237-96222024V33E2024631.EN)
Supplement: Translation

# Translation: Mortalidade por covid-19 de migrantes internacionais no Brasil: análise espaço-temporal, 2020-2022

**DOI:** 10.1590/S2237-96222024V33E2024631.PT

**Authors:** João Roberto Cavalcante, Anete Trajman, Eduardo Faerstein

**Affiliations:** 1 Universidade do Estado do Rio de Janeiro, Departamento de Epidemiologia, Rio de Janeiro, RJ, Brasil; 2 Universidade Federal do Rio de Janeiro, Departamento de Clínica Médica, Rio de Janeiro, RJ, Brasil; 3 McGill University, McGill International TB Centre, Montreal, Canadá; 4 Rede Brasileira de Pesquisa em Tuberculose, Brasil

**Keywords:** Migrantes, Covid-19, Mortalidade, Epidemiologia, Saúde Global, Brasil

## Abstract

**Objetivo:**

Descrever o perfil de óbitos e analisar a distribuição espaço-temporal da mortalidade por covid-19 em migrantes internacionais residentes no Brasil no período 2020-2022.

**Métodos:**

Trata-se estudo transversal descritivo e ecológico, com dados secundários. Foram analisadas as frequências absolutas e relativas do perfil sociodemográfico e coeficientes de mortalidade (CM). Foram calculados o excesso de risco e a autocorrelação espacial global e local.

**Resultados:**

Foram registrados 7.737 óbitos no período, com maior frequência em 2021 (3.952). O CM do Brasil foi de 515/100 mil, com CM mais altos nas macrorregiões Sudeste (751/100 mil) e Centro-Oeste (525/100 mil). O perfil de óbitos predominante foi de homens (5.041); faixa etária ≥ 81 anos (3.612); raça/cor da pele branca (5.685); casados (3.406); nascidos em Portugal (2.437). Foi identificada autocorrelação espacial global em 2022 e autocorrelação espacial local no período.

**Conclusão:**

O elevado CM indica necessidade de intervenções de políticas de saúde em regiões com alta concentração de população migrante.

## Contribuições do estudo

**Principais resultados:**

O coeficiente de mortalidade por covid-19 em migrantes, no Brasil, foi de 515/100 mil, e maior no Sudeste e no Centro-Oeste do país. O maior número de óbitos ocorreu em 2021, entre homens brancos, casados, com mais de 81 anos, vindos principalmente de Portugal.

**Implicações para os serviços:**

Este estudo recomenda a criação de políticas para migrantes internacionais no Brasil, como a priorização em campanhas de vacinação, serviços de tradução e estratégias de integração para melhorar o acesso ao sistema de saúde, a fim de diminuir óbitos.

**Perspectivas:**

O Ministério da Saúde necessita criar a Política Nacional de Saúde de Migrantes, Refugiados e Apátridas, e a variável país de nascimento/nacionalidade/natural deve se tornar de preenchimento obrigatório nos sistemas de informação em saúde.

## INTRODUÇÃO

Os processos migratórios contemporâneos são um fenômeno global, e vêm adquirindo conformações específicas nos diversos continentes.^1^ Motivado por busca por melhores condições de vida, saúde, oportunidades de trabalho ou refúgio, constitui uma dimensão complexa da dinâmica populacional atual.^1^ No âmbito global, estima-se um contingente de 281 milhões de migrantes internacionais, contabilizados até o final de 2020 – os maiores números já registrados.^2^ No Brasil, país que historicamente recebeu e ainda recebe fluxos migratórios substanciais, a presença dos atuais 1,6 milhão de migrantes residentes no território é componente importante da nossa dinâmica demográfica.^3^

Historicamente, tendo registrado grandes fluxos migratórios de portugueses, japoneses, italianos, alemães, além de pessoas escravizadas provenientes da Nigéria, Costa do Marfim, Congo, Angola e Moçambique, o Brasil registra atualmente fluxos massivos de migrantes vindos principalmente de países da América Latina e Central, como venezuelanos e haitianos.^4,5^

Migrantes internacionais em geral estão sujeitos a desafios socioeconômicos, ambientais, culturais e psicossociais que impactam seu processo saúde/doença antes, durante e depois da migração.^6,7^ Os mais vulneráveis são aqueles que migram de forma involuntária, como refugiados e apátridas.^6^ No Brasil, os principais problemas de saúde de migrantes internacionais são doenças crônicas, transtornos mentais e violências, diferentemente de outros países de acolhimento, como os da Europa, que, em sua maioria, registram atendimentos de saúde por doenças transmissíveis.^7^

Entretanto, há escassez de dados sobre o perfil de saúde dessa população.^8^ Ainda que alguns países tenham sistemas de informação em saúde robustos, que registram dados de saúde e doença da população residente há décadas, esses sistemas são insuficientes em relação aos migrantes, tornando-os invisibilizados, o que dificulta a implementação de políticas de saúde específicas que os protejam.^8^

No Brasil, apenas o Sistema de Informação sobre Mortalidade (SIM) possui registro preenchido com o dado de país de nascimento da população falecida.^9^ Ainda que os registros do SIM estejam disponíveis desde 1979, até onde sabemos esses dados nunca foram analisados e divulgados em nível nacional.^8^ Parte significativa dessa dificuldade surge da necessidade de calcular o número total de pessoas em risco (denominadores) para calcular os coeficientes de mortalidade (CM), permitindo assim a comparação entre diferentes locais de residência ou países de nascimento.^8^ Isso não foi diferente durante a pandemia de covid-19. Portanto, o número de casos e óbitos pela doença, entre migrantes internacionais residentes no Brasil, ainda é desconhecido. Talvez por este motivo, migrantes não foram considerados um grupo prioritário no plano nacional de imunização contra a doença no país.^10^

O presente estudo teve como objetivo descrever o perfil de óbitos e analisar a distribuição espaço-temporal da mortalidade por covid-19 entre migrantes internacionais residentes no Brasil, no período 2020-2022.

## MÉTODOS

Foi realizado um estudo transversal descritivo e ecológico, com dados secundários, para avaliar o perfil de óbitos e a distribuição espaço-temporal dos CM por covid-19 em migrantes internacionais residentes no Brasil. As unidades de análise do estudo ecológico foram os países de nascimento, Unidades da Federação (UF) e macrorregiões de residência no país.

O Brasil possui 5.570 municípios, distribuídos em 26 estados e no Distrito Federal, que compõem as cinco macrorregiões do país. É o quinto maior país do mundo em extensão territorial (8.547.403 km²).^11,12^ O país possuía uma população total estimada de 203.080.756 habitantes em 2022 – a sétima maior população do mundo –, com densidade demográfica de 23,8 habitantes por km².^11,12^

Foram incluídos os óbitos por covid-19 registrados entre 2020 e 2022.^13^ O ano de 2023 não foi incluído, pois os dados disponíveis estavam incompletos.^9^ Para a separação de migrantes internacionais e brasileiros nos bancos do SIM, foi utilizada a variável “NATURAL”.^9^ Foram considerados óbitos por covid-19 os registros no banco de dados do SIM, de acesso aberto do sítio do Opendatasus, do Ministério da Saúde do Brasil, cuja causa básica do óbito tenham sido o CID-10 B34.2 (infecção por coronavírus de localização não especificada) e o J98.8 (outros transtornos respiratórios especificados).^9^ O código J98.8 foi utilizado pelas equipes de vigilância em saúde do Brasil quando não foi possível encerrar os óbitos de “síndrome respiratória aguda grave (SRAG) de causa indeterminada” como covid-19, devido ao grande volume de trabalho durante a pandemia.^9,14^

A partir destes dados, foram calculadas as frequências absolutas e relativas referentes ao ano do óbito (2020, 2021 e 2022), sexo (masculino, feminino e ignorado), faixa etária (< 1, 1 a 10, 11 a 20, 21 a 30, 31 a 40, 41 a 50, 51 a 60, 61 a 70, 71 a 80, ≥ 81, ignorado), raça/cor da pele (branca, preta, parda, amarela, indígena e ignorado) e estado civil (solteiro, casado, viúvo, separado/divorciado, união estável e ignorado). Para o cálculo dos CM por 100 mil habitantes, foi necessária a elaboração de denominadores da população de migrantes residentes no Brasil, por país de nascimento, UF e macrorregiões no período do estudo (2020 a 2022). Foi necessário calcular as populações de 2011 a 2022, mesmo só se utilizando as populações de 2020 a 2022 como denominadores, pois o último dado calculado de população de migrantes internacionais no Brasil era da população do ano de 2010. Dessa forma, os dados abertos utilizados para os cálculos dos anos seguintes foram os do Censo Demográfico de 2010, do Instituto Brasileiro de Geografia e Estatística (IBGE), do Sistema de Registro Nacional Migratório (SISMIGRA), do Ministério da Justiça (2011 a 2022), além dos dados de óbitos do SIM (2011 a 2022).^9,15,16^ A partir desses dados, foi possível calcular, pela primeira vez, as populações de migrantes internacionais residentes no Brasil de 2020 a 2022. A fórmula para o cálculo dessas populações para o ano de 2011 foi:



Para as populações de 2012 a 2022, não foi necessário mais o uso do Censo de 2010 de forma separada, pois este já estava incorporado na população calculada para o ano de 2011. A fórmula utilizada para calcular as populações dos anos seguintes foi:



Foi utilizada a população do meio do período (PMP) no denominador dos CM, sendo considerada a data de 2 de julho como o meio do período de cada ano, conforme demonstrado na fórmula descrita abaixo:^17^


$$\text{CM}=\frac{\text{Número de óbitos}}{\text{População do meio do período}}\times 100.000$$

As tabelas contendo os CM foram apresentadas no formato de heatmap, em que os valores menores de cada coluna possuem uma cor mais clara e os valores maiores aparecem em cor mais escura, no intuito de facilitar a visualização. Os CM de covid-19 por UF também foram utilizados para calcular o excesso de risco, o índice de Moran Global (I de Moran), e o indicador local de associação espacial (LISA).^18^

O excesso de risco foi apresentado em mapas temáticos e calculado ao se compararem os CM por covid-19 em diferentes UF do Brasil com o CM nacional, observando se os CM em cada UF eram maiores ou menores do que o CM nacional.^18^ O I de Moran e o LISA, por sua vez, analisam a autocorrelação espacial global e local, com o objetivo de identificar padrões na distribuição espacial dos indicadores apresentados.^18^ Neste estudo, foram utilizados para identificar aglomerados de óbitos nas UF do Brasil e suas significâncias estatísticas no período do estudo.^18^ Para o cálculo do I de Moran, utilizamos uma matriz de pesos espaciais que define as relações de vizinhança entre as UFs e aplicamos testes de significância estatística para avaliar a hipótese nula de ausência de autocorrelação espacial.^18^ Para o LISA, por sua vez, desagregamos a análise global, identificando as áreas com altos e baixos CM. Este procedimento inclui o cálculo dos valores de LISA para cada UF e a avaliação de suas significâncias através de testes de permutação, permitindo a detecção de padrões espaciais locais durante o período do estudo.^18^

Os quadrantes alto-alto, baixo-baixo, alto-baixo e baixo-alto foram utilizados para classificar a autocorrelação espacial local.^18^ O quadrante alto-alto indica áreas com altos valores próximas de outras áreas com altos valores; o baixo-baixo indica áreas com baixos valores próximas de outras áreas com baixos valores; o alto-baixo indica áreas com altos valores próximas de áreas com baixos valores; e o baixo-alto indica áreas com baixos valores próximas de áreas com altos valores.^18^ Esses quadrantes foram apresentados por meio de gráficos de dispersão e mapas de *cluster*.^18^ O *software* R Studio 3.3.0+ foi utilizado para manipulação e análise de dados, e os *softwares* Quantum Geographic Information System (QGIS) versão 3.24 e GeoDa versão 1.22 foram utilizados para as análises espaciais.

Por se tratar de um estudo com dados secundários, agregados e de acesso livre, não houve necessidade de aprovação por um Comitê de Ética em Pesquisa, conforme a resolução no 510 de 2016 do Conselho Nacional de Ética em Pesquisa. Os bancos analisados eram públicos, anonimizados, conforme predito na Lei Geral de Proteção de Dados (Lei n° 13.709/2018).^19,20^

## RESULTADOS

Entre 2020 e 2022, foram registrados 7.737 óbitos por covid-19 em migrantes internacionais residentes no Brasil, sendo a maioria no ano de 2021 (Tabela 1). A maioria dos óbitos foram em indivíduos do sexo masculino e em idosos, com a média de idade de óbitos de 76,3 anos. A raça/cor da pele predominante foi a branca, seguida da amarela. A maioria dos indivíduos eram casados ou viúvos (Tabela 1).

**Tabela 1:** Perfil sociodemográfico dos óbitos de migrantes internacionais residentes no Brasil por covid-19, entre 2020 e 2022

**Características socio-demográficas**	**2020**		**2021**		**2022**		**2020-2022 (Período completo)**	
	**N**	**%**	**N**	**%**	**N**	**%**	**N**	**%**
**Ano do óbito**	2.865	37,0	3.952	51,1	920	11,9	7.737	100
**Sexo**								
Masculino	1.871	65,3	2.617	66,2	553	60,1	5.041	71,5
Feminino	993	34,7	1.333	33,7	367	39,9	2.693	38,2
Ignorado	1	0,03	2	0,1	0	0,0	3	0,0
**Faixa etária (anos)**								
< 1	0	0,0	2	0,1	0	0,0	2	0,0
1 a 10	1	0,0	1	0,0	2	0,2	4	0,1
11 a 20	3	0,1	4	0,1	1	0,1	8	0,1
21 a 30	15	0,5	39	1,0	2	0,2	56	0,7
31 a 40	54	1,9	131	3,3	11	1,2	196	2,5
41 a 50	73	2,5	244	6,2	11	1,2	328	4,2
51 a 60	160	5,6	357	9,0	15	1,6	532	6,9
61 a 70	376	13,1	655	16,6	69	7,5	1.100	14,2
71 a 80	695	24,3	1.025	25,9	179	19,5	1.899	24,5
≥ 81	1.488	51,9	1.494	37,8	630	68,5	3.612	46,7
Ignorado	0	0,0	0	0,0	0	0,0	0	0,0
**Raça/cor da pele**								
Branca	2.127	74,2	2.845	72,0	713	77,5	5.685	73,5
Amarela	329	11,5	630	15,9	70	7,6	1.029	13,3
Parda	279	9,7	269	6,8	105	11,4	653	8,4
Preta	68	2,4	101	2,6	14	1,5	183	2,4
Indígena	15	0,5	19	0,5	4	0,4	38	0,5
Ignorado	47	1,6	88	2,2	14	1,5	149	1,9
**Estado civil**								
Solteiro(a)	301	10,5	571	14,4	100	10,9	972	12,6
Casado(a)	1.229	42,9	1.818	46,0	359	39,0	3.406	44,0
Viúvo(a)	942	32,9	1.014	25,7	359	39,0	2.315	29,9
Separado(a)/divorciado(a)	174	6,1	261	6,6	53	5,8	488	6,3
União estável	86	3,0	117	3,0	17	1,8	220	2,8
Ignorado	133	4,6	171	4,3	32	3,5	336	4,3

Em relação ao país de nascimento, a maioria era de Portugal, seguido de Itália, Japão, Bolívia, Espanha, Venezuela, Alemanha, Paraguai, Peru, Líbano, entre outros (Tabela 2). Os maiores CM por covid-19 foram de migrantes internacionais nascidos na Lituânia, seguida de Jordânia, Portugal, Hungria, Líbano, Egito, Polônia, Japão, Grécia, Itália, entre outros (Tabela 2).

**Tabela 2:** Distribuição dos óbitos de migrantes internacionais residentes no Brasil por covid-19 por país de nascimento, entre 2020 e 2022

País de nascimento	2020				2021				2022				2020-2022 (Período completo)			
	N	%	PMP	CM	N	%	PMP	CM	N	%	PMP	CM	N	%	PMP	CM
Alemanha	37	1,3	27.505,0	134,5	175	4,4	27.505,5	636,2	30	3,3	27.682,5	108,4	242	3,1	27.505,5	879,8
Angola	14	0,5	15.961,5	87,7	20	0,5	16.834,5	118,8	1	0,1	18.597,5	5,4	35	0,5	16.834,5	207,9
Argentina	62	2,2	64.561,0	96,0	109	2,8	67.330,5	161,9	17	1,8	71.364,5	23,8	188	2,4	67.330,5	279,2
Áustria	7	0,2	2.690,5	260,2	15	0,4	2.696,5	556,3	5	0,5	2.746,0	182,1	27	0,3	2.696,5	1.001,3
Bélgica	4	0,1	4.143,5	96,5	8	0,2	4.202,0	190,4	0	0,0	4.287,5	0,0	12	0,2	4.202,0	285,6
Bolívia	177	6,2	105.624,0	167,6	268	6,8	109.777,0	244,1	38	4,1	115.422,5	32,9	483	6,2	109.777,0	440,0
Chile	63	2,2	22.885,5	275,3	104	2,6	23.307,0	446,2	17	1,8	23.846,5	71,3	184	2,4	23.307,0	789,5
China	54	1,9	50.808,5	106,3	105	2,7	51.552,0	203,7	16	1,7	52.524,0	30,5	175	2,3	51.552,0	339,5
Colômbia	24	0,8	69.417,0	34,6	53	1,3	73.210,5	72,4	6	0,7	77.639,0	7,7	83	1,1	73.210,5	113,4
Coréia do Sul	22	0,8	19.586,0	112,3	32	0,8	19.985,5	160,1	3	0,3	20.388,0	14,7	57	0,7	19.985,5	285,2
Cuba	10	0,3	31.040,0	32,2	29	0,7	31.729,5	91,4	1	0,1	33.718,0	3,0	40	0,5	31.729,5	126,1
Egito	19	0,7	1.997,0	951,4	26	0,7	2.008,0	1.294,8	6	0,7	2.028,5	295,8	51	0,7	2.008,0	2.539,8
Equador	6	0,2	8.602,5	69,7	7	0,2	9.094,5	77,0	1	0,1	9.976,5	10,0	14	0,2	9.094,5	153,9
Espanha	188	6,6	35.427,0	530,7	207	5,2	34.755,0	595,6	68	7,4	34.253,5	198,5	463	6,0	34.755,0	1.332,2
EUA	8	0,3	51.346,5	15,6	25	0,6	52.823,0	47,3	10	1,1	54.898,5	18,2	43	0,6	52.823,0	81,4
França	16	0,6	32.583,0	49,1	27	0,7	33.397,0	80,8	7	0,8	34.673,5	20,2	50	0,6	33.397,0	149,7
Grécia	13	0,5	2.304,0	564,2	29	0,7	2.256,0	1.285,5	3	0,3	2.214,5	135,5	45	0,6	2.256,0	1.994,7
Guiana	4	0,1	1.045,5	382,6	12	0,3	1.065,0	1.126,8	1	0,1	1.120,0	89,3	17	0,2	1.065,0	1.596,2
Haiti	32	1,1	154.658,0	20,7	38	1,0	165.342,0	23,0	7	0,8	170.067,0	4,1	77	1,0	165.342,0	46,6
Holanda	6	0,2	8.034,5	74,7	16	0,4	8.139,0	196,6	4	0,4	8.308,0	48,1	26	0,3	8.139,0	319,4
Hungria	7	0,2	679,5	1.030,2	11	0,3	668,0	1.646,7	2	0,2	657,0	304,4	20	0,3	668,0	2.994,0
Israel	6	0,2	1.515,0	396,0	9	0,2	1.532,0	587,5	3	0,3	1.538,5	195,0	18	0,2	1.532,0	1.174,9
Itália	274	9,6	39.473,0	694,1	338	8,6	38.617,0	875,3	81	8,8	37.981,5	213,3	693	9,0	38.617,0	1.794,5
Japão	274	9,6	30.555,5	896,7	252	6,4	29.160,0	864,2	120	13,0	28.046,0	427,9	646	8,3	29.160,0	2.215,4
Jordânia	6	0,2	538,0	1.115,2	17	0,4	523,5	3.247,4	1	0,1	513,0	194,9	24	0,3	523,5	4.584,5
Líbano	67	2,3	7.092,0	944,7	115	2,9	7.010,0	1.640,5	19	2,1	7.015,0	270,8	201	2,6	7.010,0	2.867,3
Lituânia	5	0,2	238,5	2.096,4	3	0,1	198,0	1.515,2	3	0,3	168,5	1.780,4	11	0,1	198,0	5.555,6
Moçambique	7	0,2	4.028,0	173,8	7	0,2	4.171,5	167,8	2	0,2	4.405,0	45,4	16	0,2	4.171,5	383,6
Nigéria	5	0,2	4.833,0	103,5	6	0,2	5.010,0	119,8	1	0,1	5.276,5	19,0	12	0,2	5.010,0	239,5
Paraguai	50	1,7	47.852,0	104,5	142	3,6	49.596,0	286,3	29	3,2	52.562,0	55,2	221	2,9	49.596,0	445,6
Peru	85	3,0	40.400,5	210,4	123	3,1	41.570,5	295,9	9	1,0	43.183,0	20,8	217	2,8	41.570,5	522,0
Polônia	41	1,4	3.982,5	1.029,5	34	0,9	3.923,5	866,6	18	2,0	3.938,0	457,1	93	1,2	3.923,5	2.370,3
Portugal	1.005	35,1	86.271,0	1.164,9	1.132	28,6	81.119,5	1.395,5	300	32,6	76.359,5	392,9	2.437	31,5	81.119,5	3.004,2
Reino Unido	3	0,1	14.538,0	20,6	14	0,4	14.862,5	94,2	3	0,3	15.314,5	19,6	20	0,3	14.862,5	134,6
Romênia	17	0,6	2.502,0	679,5	12	0,3	2.486,5	482,6	7	0,8	2.499,5	280,1	36	0,5	2.486,5	1.447,8
Rússia	7	0,2	4.773,5	146,6	5	0,1	5.090,5	98,2	3	0,3	5.624,5	53,3	15	0,2	5.090,5	294,7
Síria	19	0,7	4.115,5	461,7	20	0,5	4.027,0	496,6	5	0,5	3.950,5	126,6	44	0,6	4.027,0	1.092,6
Suíça	7	0,2	6.150,0	113,8	7	0,2	6.252,0	112,0	7	0,8	6.441,5	108,7	21	0,3	6.252,0	335,9
Turquia	2	0,1	1.452,0	137,7	7	0,2	1.537,0	455,4	1	0,1	1.678,5	59,6	10	0,1	1.537,0	650,6
Uruguai	41	1,4	48.108,5	85,2	88	2,2	49.169,5	179,0	22	2,4	50.682,5	43,4	151	2,0	49.169,5	307,1
Venezuela	95	3,3	221.963,5	42,8	205	5,2	272.560,0	75,2	23	2,5	349.267,5	6,6	323	4,2	272.560,0	118,5
Outros	76	2,7	137.441,5	55,3	100	2,5	140.832,5	71,0	20	2,2	146.056,5	13,7	196	2,5	140.832,5	139,2
Total	2.865	100	1.418.723,5	199,6	3.952	100	1.496.927	260,9	920	100	1.608.915	56,5	7.737	100	1.496.927	516,9

Notas: N = frequência absoluta de óbitos % = frequência relativa de óbitos PMP = população do meio do período CM = coeficiente de mortalidade EUA = Estados Unidos da América Escala de cor (valores menores para valores maiores):

O CM geral foi de 515,6 óbitos por 100 mil habitantes. A macrorregião Sudeste teve o maior número de óbitos, seguida pela macrorregião Sul. Com os CM, a macrorregião Sudeste permanece em destaque, mas seguida pela macrorregião Centro-Oeste. Embora o maior número de óbitos tenha ocorrido em São Paulo e no Rio de Janeiro, os maiores CM foram observados no Acre, seguido por Goiás, São Paulo, Tocantins, entre outros (Tabela 3).

**Tabela 3:** Distribuição dos óbitos de migrantes internacionais residentes no Brasil por covid-19, por Unidade da Federação e macrorregião, entre 2020 e 2022

País de nascimento	2020				2021				2022				2020-2022 (Período completo)			
	N	%	PMP	CM	N	%	PMP	CM	N	%	PMP	CM	N	%	PMP	CM
Norte	177	6,2	210.849,5	83,9	291	7,4	241.737,0	120,4	22	2,4	288.302,0	7,6	490	6,3	241.737,0	202,7
Acre	6	0,2	3.200,5	187,5	26	0,7	3.320,0	783,1	2	0,2	3.566,0	56,1	34	0,4	3.320,0	1.024,1
Amapá	2	0,1	2.053,0	97,4	2	0,1	2.139,0	93,5	0	0,0	2.243,5	0	4	0,1	2.139,0	187,0
Amazonas	68	2,4	61.797,0	110,0	104	2,6	68.717,0	151,3	4	0,4	77.110,0	5,2	176	2,3	68.717,0	256,1
Pará	22	0,8	10.801,0	203,7	28	0,7	11.274,0	248,4	4	0,4	11.927,5	33,5	54	0,7	11.274,0	479,0
Rondônia	24	0,8	9.730,0	246,7	29	0,7	10.521,5	275,6	5	0,5	11.659,5	42,9	58	0,7	10.521,5	551,3
Roraima	50	1,7	121.722,5	41,1	97	2,5	144.160,0	67,3	6	0,7	180.108,5	3,3	153	2,0	144.160,0	106,1
Tocantins	5	0,2	1.545,5	323,5	5	0,1	1.605,5	311,4	1	0,1	1.687,0	59,3	11	0,1	1.605,5	685,1
Nordeste	94	3,3	111.290,0	84,5	186	4,7	115.096,0	161,6	41	4,5	119.999,5	34,2	321	4,1	115.096,0	278,9
Alagoas	3	0,1	3.214,5	93,3	4	0,1	3.370,5	118,7	2	0,2	3.578,0	55,9	9	0,1	3.370,5	267,0
Bahia	35	1,2	33.011,5	106,0	70	1,8	34.111,0	205,2	16	1,7	35.489,5	45,1	121	1,6	34.111,0	354,7
Ceará	17	0,6	25.333,0	67,1	33	0,8	26.061,0	126,6	4	0,4	27.177,5	14,7	54	0,7	26.061,0	207,2
Maranhão	4	0,1	7.239,0	55,3	14	0,4	7.497,0	186,7	1	0,1	7.808,5	12,8	19	0,2	7.497,0	253,4
Paraíba	3	0,1	6.490,5	46,2	9	0,2	6.705,5	134,2	1	0,1	6.990,5	14,3	13	0,2	6.705,5	193,9
Pernambuco	23	0,8	19.773,5	116,3	35	0,9	20.400,5	171,6	13	1,4	21.205,0	61,3	71	0,9	20.400,5	348,0
Piauí	1	0,0	2.676,0	37,4	3	0,1	2.809,0	106,8	0	0,0	2.941,5	0	4	0,1	2.809,0	142,4
Rio Grande do Norte	6	0,2	10.602,0	56,6	9	0,2	11.081,5	81,2	3	0,3	11.613,5	25,8	18	0,2	11.081,5	162,4
Sergipe	2	0,1	2.950,0	67,8	9	0,2	3.060,0	294,1	1	0,1	3.195,5	31,3	12	0,2	3.060,0	392,2
Centro-Oeste	97	3,4	87.989,5	110,2	346	8,8	93.679,5	369,3	49	5,3	100.918,0	48,6	492	6,4	93.679,5	525,2
Distrito Federal	31	1,1	30.672,5	101,1	56	1,4	31.815,5	176,0	12	1,3	33.289,5	36,0	99	1,3	31.815,5	311,2
Goiás	19	0,7	18.291,5	103,9	154	3,9	19.238,0	800,5	8	0,9	20.251,0	39,5	181	2,3	19.238,0	940,8
Mato Grosso	17	0,6	17.221,0	98,7	37	0,9	18.673,5	198,1	5	0,5	20.522,5	24,4	59	0,8	18.673,5	316,0
Mato Grosso do Sul	30	1,0	21.804,5	137,6	99	2,5	23.952,5	413,3	24	2,6	26.855,0	89,4	153	2,0	23.952,5	638,8
Sudeste	2.275	79,4	731.416,5	311,0	2.651	67,1	746.130,5	355,3	682	74,1	769.095,5	88,7	5.608	72,5	746.130,5	751,6
Espírito Santo	14	0,5	13.405,5	104,4	17	0,4	13.779,5	123,4	6	0,7	14.233,5	42,2	37	0,5	13.779,5	268,5
Minas Gerais	32	1,1	56.649,5	56,5	109	2,8	59.054,0	184,6	30	3,3	62.209,0	48,2	171	2,2	59.054,0	289,6
Rio de Janeiro	646	22,5	167.520,0	385,6	722	18,3	168.631,5	428,2	144	15,7	171.568,5	83,9	1512	19,5	168.631,5	896,6
São Paulo	1.583	55,3	493.841,5	320,5	1.803	45,6	504.665,5	357,3	502	54,6	521.084,5	96,3	3.888	50,3	504.665,5	770,4
Sul	222	7,7	280.526,5	79,1	478	12,1	303.910,0	157,3	126	13,7	335.853,0	37,5	826	10,7	303.910,0	271,8
Paraná	107	3,7	99.427,0	107,6	273	6,9	108.051,5	252,7	75	8,2	119.965,5	62,5	455	5,9	108.051,5	421,1
Rio Grande do Sul	59	2,1	95.603,0	61,7	112	2,8	101.554,0	110,3	26	2,8	109.236,5	23,8	197	2,5	101.554,0	194,0
Santa Catarina	56	2,0	85.496,5	65,5	93	2,4	94.304,5	98,6	25	2,7	106.651,0	23,4	174	2,2	94.304,5	184,5
Brasil	2.865	100	1.422.072	201,5	3.952	100	1.500.553	263,4	920	100	1.614.168	57,0	7.737	100	1.500.553	515,6

Notas: N = frequência absoluta de óbitos % = frequência relativa de óbitos PMP = população do meio do período CM = coeficiente de mortalidade Escala de cor (valores menores para valores maiores):

O excesso de risco de mortalidade em 2020 foi maior no Pará, no Rio de Janeiro, em Rondônia, em São Paulo e no Tocantins, que possuíam o CM até duas vezes maior que o CM do Brasil (Figura 1). Já em 2021, destacam-se o Acre e Goiás, e em 2022, Mato Grosso do Sul, Paraná, Pernambuco, Rio de Janeiro, São Paulo e Tocantins, com o CM até duas vezes maior que o CM geral do Brasil. Em 2022, nenhum estado apresentou CM mais de duas vezes acima do CM geral do Brasil (Figura 1).

**Figura 1:** Coeficientes e excesso de risco da mortalidade por covid-19 em migrantes internacionais residentes no Brasil, por Unidade da Federação, entre 2020 e 2022 (N = 7.737 óbitos))

O I de Moran indicou autocorrelação espacial global em 2022, sugerindo que os óbitos por covid-19 estariam relacionados entre si e agrupados em diferentes estados do país, formando clusters (I de Moran 0,338; p-valor=0,007). O LISA, por sua vez, indicou autocorrelação espacial local. Entre 2020 e 2021, Mato Grosso e Minas Gerais apresentaram CM mudando de valores baixos para altos (baixo-alto), o que sugere um aumento significativo nas taxas de mortalidade por covid-19 nessas áreas. Entre 2021 e 2022, Mato Grosso do Sul e, especificamente em 2022, os estados do Paraná, Minas Gerais, Rio de Janeiro e São Paulo mostraram CM altos que aumentaram ainda mais (alto-alto) (Figura 2).

**Figura 2:** Autocorrelação espacial global (I de Moran) e local (LISA) dos coeficientes de mortalidade por covid-19 em migrantes internacionais residentes no Brasil, por Unidade da Federação, entre 2020 e 2022 (N = 7.737 óbitos)

## DISCUSSÃO

Até onde foi possível identificar, este foi o primeiro estudo em nível nacional a calcular os CM de migrantes internacionais residentes no Brasil. Encontramos um CM por covid-19 quase duas vezes maior entre migrantes do que na população geral, sugerindo um elevado risco de letalidade nesta população. O perfil dos migrantes internacionais residentes no Brasil que faleceram por covid-19, entre 2020 e 2022, foi similar ao da população geral,^21^ com maior número de óbitos em 2021, em idosos, do sexo masculino, vindos principalmente de países europeus e concentrados nas macrorregiões Sudeste e Sul.

O Brasil teve alta mortalidade por covid-19 em números absolutos – a segunda maior no mundo, com pico de óbitos durante a transmissão da variante Gama no primeiro semestre de 2021.^22^ O maior número de óbitos em idosos não surpreende; a idade avançada foi rapidamente reconhecida como um fator de risco para alta letalidade por covid-19 na população geral.^23^ Houve migrações internacionais em massa de Portugal, Itália e Japão para o Brasil durante a primeira metade do século XX,^1,8^ e estes migrantes se encontram na faixa etária mais elevada, o que pode justificar a origem dos migrantes que mais faleceram por covid-19. Em estudos realizados na França e na Espanha, a mortalidade por covid-19 foi maior entre migrantes idosos não europeus.^24,25^

Em outro único estudo encontrado sobre a mortalidade de migrantes internacionais, realizado na Itália e publicado em 2020, a maioria dos migrantes eram nascidos principalmente na Albânia, França, Índia, Líbia e Romênia. Esses migrantes possuíam perfil sociodemográfico similar ao da população nacional, sendo a única diferença que os migrantes que mais evoluíram para óbito, ainda que idosos, eram mais novos que a população nascida no país^26^ .

As macrorregiões Sudeste e Sul concentraram 83,2% do total de óbitos, 75,7% deles nos estados de São Paulo, Rio de Janeiro e Paraná. Esses locais também concentram a maioria da população de migrantes internacionais residentes no Brasil.^16^ Entretanto, os CM maiores foram observados nas macrorregiões Sudeste e Centro-Oeste. Os estados do Acre e de Goiás se destacam, principalmente no ano de 2021. O Acre tem recebido diversos migrantes internacionais nos últimos anos, principalmente haitianos.^5^ O CM alto de Goiás pode ser explicado pelo elevado número de migrantes internacionais idosos que chegaram da Alemanha para essa UF durante o século XX, para trabalharem nas plantações de uva.^27^

Atualmente, mesmo com a Constituição Federal de 1988, a Lei Orgânica da Saúde de 1990 e a Lei de Migração de 2017, que garante acesso à saúde e proteção aos migrantes internacionais no Brasil, ainda há dificuldade para esta população realizar atendimentos e tratamentos.^7^ Alguns movimentos positivos têm se realizado no âmbito da saúde dos migrantes internacionais em anos recentes no Brasil, com o retorno do país ao Pacto Global para Migração Segura, Ordenada e Regular, e a publicação, pelo Ministério da Saúde, da portaria que cria um grupo de trabalho que colabore para a criação da futura Política Nacional de Saúde dos Migrantes, Refugiados e Apátridas.^28,29^

Nosso estudo apresenta algumas limitações. Como não há registros oficiais do perfil da população de migrantes residentes no Brasil, não foi possível comparar os CM por covid-19 com o perfil geral de migrantes do país, para estimar se o maior número de óbitos se deve à maior população destes migrantes ou ao maior risco nestas subpopulações. No âmbito da covid-19, essa população foi invisibilizada no E-SUS Notifica e no Sistema de Informações sobre Vigilância Epidemiológica da Gripe (SIVEP-Gripe), pois, em ambos os sistemas, as variáveis “país de nascimento, “naturalidade” ou “nacionalidade” não são de preenchimento obrigatório. No E-SUS Notifica, um outro fator faz com que esses dados sejam perdidos, pois a variável “país de nascimento” só permite o preenchimento nos casos em que a pessoa não tem Cadastro de Pessoa Física (CPF). No entanto, migrantes internacionais documentados podem obter CPF no Brasil, o que torna o SIM o único sistema de informações com dados desses indivíduos.^8^

O Censo de 2020 do IBGE não foi realizado, e o Censo de 2022 não teve os resultados liberados sobre a população migrante até o final da execução desta pesquisa, o que inviabilizou estimativas mais precisas dos denominadores para cálculo dos CM. A falta de dados, por exemplo, de migrantes internacionais indocumentados no Brasil, e dados incompletos do SISMIGRA (2011-2022) impossibilitou a criação de denominadores mais precisos e que considerassem o sexo e faixa etária dessas populações, importantes para melhor avaliação e comparação de CM. Também não foi possível realizar interpolação linear para as populações de migrantes por municípios, devido ao deslocamento dos endereços de residências que eles fazem constantemente entre cidades próximas. Tentamos minimizar essa limitação, ao elaborar análises por macrorregiões e UF, pois são unidades de análises maiores, que diminuem a possibilidade de viés.

Concluímos que a invisibilidade dos migrantes internacionais nas estatísticas nacionais de saúde, durante a pandemia de covid-19, reflete uma lacuna relevante na inclusão deste grupo nas políticas de saúde pública. O Brasil precisa criar com urgência políticas de saúde para migrantes internacionais, inserindo-os como população vulnerável e prioritária nos planos de emergência, que potencializem o acesso dessas pessoas aos serviços de saúde, a medicamentos e a insumos como vacinas, assim garantindo a melhoria das suas condições de saúde no longo prazo.
